# Expression, regulating mechanism and therapeutic target of KIF20A in multiple cancer

**DOI:** 10.1016/j.heliyon.2023.e13195

**Published:** 2023-01-25

**Authors:** Zheng Jin, Fei Peng, Chao Zhang, Shuang Tao, Damo Xu, Zhenhua Zhu

**Affiliations:** a Department of Respirology & Allergy, The Third Affiliated Hospital of Shenzhen University, Shenzhen, Guangdong Province, China; b Department of Medicine, Division of Endocrinology, Diabetes and Metabolism, Baylor College of Medicine, Houston, Texas, USA; c Guangzhou Women and Children's Medical Center, Guangdong Provincial Clinical Research Center for Child Health, Guangzhou Medical University, Guangzhou, Guangdong Province, China; d Department of Otorhinolaryngology Head and Neck Surgery, Longgang Central Hospital of Shenzhen, Guangzhou University of Chinese Medicine, Guangzhou, Guangdong Province, China; e State Key Laboratory of Respiratory Disease for Allergy at Shenzhen University, Shenzhen Key Laboratory of Allergy and Immunology, Shenzhen University School of Medicine, Shenzhen, Guangdong Province, China; f Department of Orthopaedic Trauma, The Third Affiliated Hospital of Southern Medical University, Guangzhou, Guangdong Province, China

**Keywords:** KIF20A, Cancer, Expression, Regulating mechanisms, Therapeutic target, ATP, adenosine triphosphate, BTC, biliary tract cancer, Cdk1, cyclin-dependent kinase 1, circRNA, circular RNA, CPC, chromosomal passenger complex, CTL, cytotoxic T lymphocyte, DLG5, discs large MAGUK scaffold protein 5, EMT, epithelial-mesenchymal transition, FoxM1, forkhead box protein M1, GC, gastric cancer, GEM, gemcitabine, Gli2, glioma-associated oncogene 2, HLA, human leukocyte antigen, HNMT, head-and-neck malignant tumor, IRF, interferon regulatory factor, JAK, Janus kinase, KIF20A, kinesin family member 20A, LP, long peptide, Mad2, mitotic arrest deficient 2, MHC I, major histocompatibility complex I, miRNA, microRNA, MKlp2, mitotic kinesin-like protein 2, OS, overall survival, PBMC, peripheral blood mononuclear cell, Plk1, polo-like kinase 1

## Abstract

Kinesin family member 20A (KIF20A) is a member of the kinesin family. It transports chromosomes during mitosis, plays a key role in cell division. Recently, studies proved that KIF20A was highly expressed in cancer. High expression of KIF20A was correlated with poor overall survival (OS). In this review, we summarized all the cancer that highly expressed KIF20A, described the role of KIF20A in cancer. We also organized phase I and phase II clinical trials of KIF20A peptides vaccine. All results indicated that KIF20A was a promising therapeutic target for multiple cancer.

## 1 Introduction

Kinesins were first identified in 1985 [1]. A total of 45 kinesin superfamily proteins (KIFs) are found in humans. They are divided into 14 subfamilies according to structure [2]. KIFs play an important role in intracellular transport. They not only transport membrane organelles, protein complexes and mRNAs to maintain cell activities, but also transport chromosomes during mitosis. They are essential for cell morphogenesis and survival [3].

Kinesins regulate mitosis precisely to ensure that mitosis occurred in order. Increased mitotic kinesin caused excessive spindle separation, premature formation and separation of sister chromatids, led to abnormal DNA distribution and aneuploidy, increased cancer occurrence and development [4,5]. KIFs were highly expressed in multiple cancer and can be used as diagnostic and prognostic factors. For example, KIF2A was highly expressed in breast cancer tissue, increased KIF2A indicated a poor OS (OR = 16.55) and lymph node metastasis [6].

As a member of KIFs, KIF20A (also known as mitotic kinesin-like protein 2, MKlp2) was increased in cancer, including glioma, and could work as an independent prognostic factor [7]. More and more studies focus on the relationship between KIF20A and cancer. In this review, we summarized the expression of KIF20A in tumors and the mechanisms of KIF20A regulating cancer, organized all clinical trials related to KIF20A. In summary, KIF20A was a potential cancer therapeutic target.

## 2 Structure and function of KIF20A

**Fig. 1:**
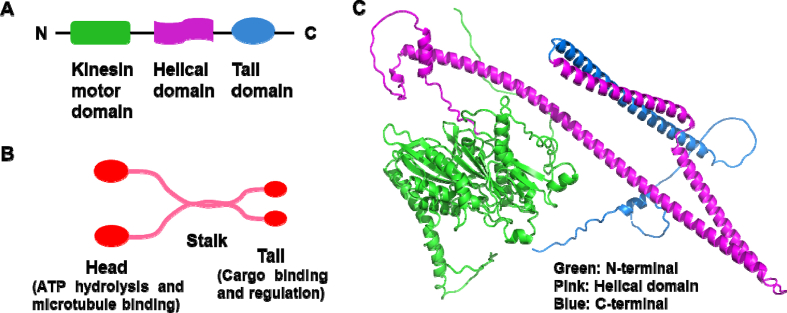
The structure of KIF20A. A. KIF20A consists of three functional domains: a N-terminal motor domain that mediates motor activity, a central helical domain containing Rab6 and myosin II binding domain, which is essential for dimerization and interaction with partner proteins, and a C-terminal tail domain that contributes to vesicle transport and interaction with partner proteins. B. N-terminal motor domain interacts with microtubules at the presence of ATP, ATP provides energy to force KIF20A move toward to the positive end of the microtubule. C-terminal tail domain contributes to vesicle transport and interaction with cargo. C. 3D structure of KIF20A. Structure data was extracted from RCSB PDB (https://www.rcsb.org/).

As early as in 1998, a kinesin like protein related to Rab6 (RB6K) was found [8]. RB6K located in the middle of spindle and mitotic groove, accumulated in mitotic cells [9]. This protein is now named KIF20A, and classified into the Kinesin-6 of kinesin superfamily. Human *KIF20A* gene is located in band q31.2 of chromosome 5 [10], encodes 890 amino acids [11]. KIF20A consists of three functional domains. N-terminal motor domain interacts with microtubules at the presence of adenosine triphosphate (ATP), ATP provides energy to force KIF20A move toward to the positive end of the microtubule [10,12]. Because of N-terminal motor domain, KIF20A is identified as an N-kinesin [13]. Helical domain could bind to Rab6 and myosin II-binding domains, which is essential for dimerization and interaction with partner proteins. C-terminal tail domain contributes to vesicle transport and interaction with cargo [14] (Fig. 1A–C).

**Fig. 2:**
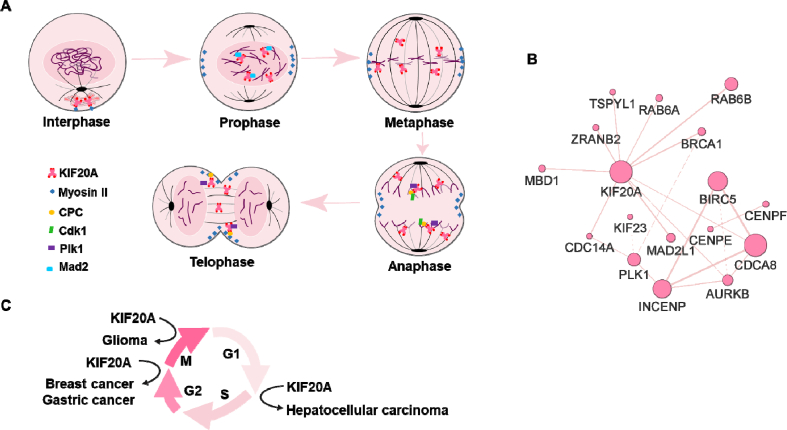
KIF20A in mitosis and tumor proliferation. A. KIF20A participates in cell division. During the interphase of the cell cycle, KIF20A co-located with Myosin II at the Golgi division hotspot to regulate the division process of Rab6-positive vesicles. In prophase, KIF20A accumulated in the nucleus, then Myosin II recruited KIF20A into the equatorial cortex before entry furrow ingression. In metaphase, as the nucleus disappears, KIF20A appears in the cytoplasm. In anaphase, chromosomal passenger complex (CPC: complex of Aurora B, INCENP, survivin and borealin) and KIF20A form a complex, which is located in the cleavage furrow and promotes the formation of cleavage furrow, and this process is mediated by decreased Cdk1 activity. In late mitosis, KIF20A mediates spindle formation. Plk1 form a complex with KIF20A and phosphorylates it, thus regulates the binding of KIF20A to microtubules. Phosphorylation of KIF20A by Plk1 is necessary for the spatial restriction of Plk1 to the central spindle during anaphase and telophase. Mad2 is a negative regulator of KIF20A. At early stage of mitosis, Mad2 negatively regulates the load of KIF20A onto the mitotic spindle. At late stage of mitosis, the localization of KIF20A at the cytokinesis of CPC is also reduced by Mad2. B. Proteins that interact with KIF20A, only physical interactions were preserved. The interaction data was extracted from GENEMANIA (https://genemania.org/). C. In glioma cancer, KIF20A contributed cytokinesis and generation of binucleated cells; in breast cancer and gastric cancer, KIF20A arrested cells at the G2/M phase; in hepatocellular carcinoma, KIF20A affected cell transition from G1 to S phase through E2F1.

KIF20A participates in cell division. During the interphase of the cell cycle, KIF20A co-located with Myosin II at the Golgi division hotspot to regulate the division process of Rab6-positive vesicles [8]. In prophase, KIF20A accumulated in the nucleus, then Myosin II recruited KIF20A into the equatorial cortex before entry furrow ingression [15]. In metaphase, as the nucleus disappears, KIF20A appears in the cytoplasm [15]. In anaphase, chromosomal passenger complex (CPC: complex of Aurora B, INCENP, survivin and borealin) and KIF20A form a complex, which is located in the cleavage furrow and promotes the formation of cleavage furrow, and this process is mediated by decreased cyclin-dependent kinase 1 (Cdk1) activity [15]. In late mitosis, KIF20A mediates spindle formation (Fig. 2A). Polo-like kinase 1 (Plk1) form a complex with KIF20A and phosphorylates it, thus regulates the binding of KIF20A to microtubules. Phosphorylation of KIF20A by Plk1 is necessary for the spatial restriction of Plk1 to the central spindle during anaphase and telophase [16]. MKlp1 (KIF23) is a kind of mitotic motor which contributes to microtubule activities, its functions are same as KIF20A [17]. It co-located with KIF20A at the central spindle and midbody, but no correlation between them [18]. Moreover, KIF20A is more important at late stage of cytokinesis [19]. Mitotic arrest deficient 2 (Mad2) is a negative regulator of KIF20A. At early stage of mitosis, Mad2 negatively regulates the load of KIF20A onto the mitotic spindle. At late stage of mitosis, the localization of KIF20A at the cytokinesis of CPC is also regulated by Mad2 [20]. We also predicted the proteins which work with KIF20A by using GENEMANIA (Fig. 2B), AURKB (Aurora B) and INCENP were included.

### 2.1 High expression of KIF20A and its role in cancer

**Fig. 3:**
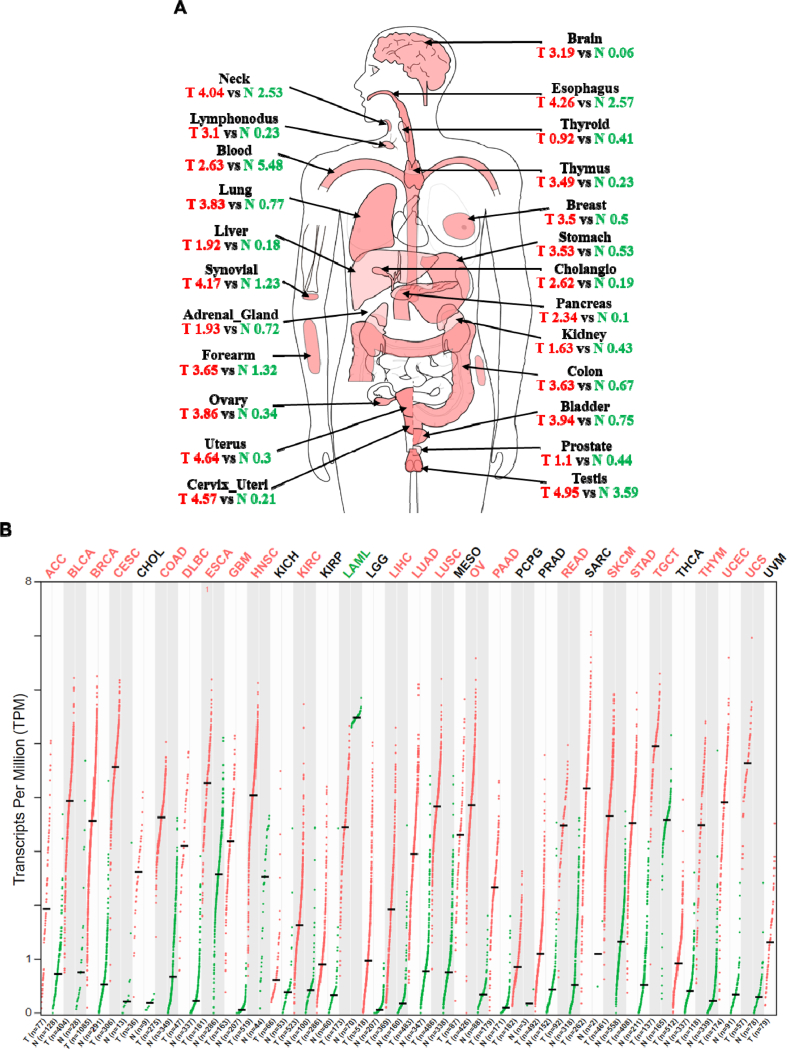
The expression of KIF20A in normal tissues and cancers. Expression results were generated with GEPIA 2 (http://gepia2.cancer-pku.cn/).

KIF20A was highly expressed in almost all of the cancer, including melanoma [21], hepatocellular carcinoma [22], esophageal squamous cell carcinoma [23] (Table 1). KIF20A was overexpressed in bladder [24] and breast cancer tissues [25]; after suppressing KIF20A, the growth of cancer cells was inhibited. This effect occurred in other tumors (Table 1). Meanwhile, KIF20A was associated with invasion, lymphatic node metastasis, distant metastasis, and tumor-node-metastasis (TNM) stage in colorectal cancer [26]. In glioma, KIF20A promoted cell proliferation and invasion [7]; down-regulated KIF20A reduced cell proliferation, induced apoptosis [27]. In hepatocellular carcinoma (HCC), inhibition of KIF20A reduced HCC growth [28]; increased KIF20A promoted both normal and pathologic hepatocyte proliferation and was related to tumor aggressiveness [29]. We used GEPIA (Fig. 3A and B) to explore the expression of KIF20A in normal and cancer tissues, results shows that KIF20A highly expressed on almost all kinds of cancers. In summary, KIF20A was highly expressed in tumors, promoted proliferation, migration, and invasion of cancer.

**Table 1:** The expression and function of KIF20A in tumors.

Tumors	Cells	Tissues	Identification method	Relationship to tumor	Mechanism	Reference
Bladder cancer	T24, BIU87, EJ, 5637, SV-HUC-1	bladder cancer tissues	qRT-PCR, WB, IHC	Overexpressed in bladder cancer tissues and cell lines.	ND	[24]
				High expression was associated with poor disease-free survival.		
				Knockdown of KIF20A inhibited the proliferation and invasion of bladder cancer cells.		
Breast cancer	T-47D, ZR-75-1, MCF-7, AU565, SK-BR-3, HCC1599, HCC1143, HCC1937, MDA-MB-231, BT-20, MDA-MB-468	breast cancer tissues	qRT-PCR, WB, IHC	High expression was associated with poor prognosis and was an independent prognostic factor.	Through regulating cell cycle arrest at the G2/M phase and subsequent mitotic cell death.	[25]
				Suppression endogenous KIF20A inhibited breast cancer cell growth.		
Cervical cancer	HeLa, SiHa	ND	ND	Circ_0005576 promotes cervical cancer cell proliferation and metastasis through KIF20A	Circ_0005576 promoted cervical cancer cell proliferation, migration and invasion as a sponge for miR-153-3p, and KIF20A was a direct target of miR-153-3p.	[50]
Cervical cancer	ND	ND	ND	UCA1 upregulated KIF20A expression to promote cervical cancer cell proliferation and invasion.	UCA1 upregulated KIF20A expression to exacerbate cervical cancer via sponging miR-204.	[57]
Cervical squamous cell carcinoma	HeLa, ME-180, HeLa229, SiHa, CasKi, HCC94, MS751, C33A	cervical cancer tissues	qRT-PCR, WB, IHC	Overexpressed in cervical cancer tissues and cell lines.	ND	[30]
				High expression was associated with poor survival and prognosis.		
Colorectal cancer	HCT-116, CACO2, RKO, LOVO, SW480, SW620, N18	CRC tissues	qRT-PCR, WB, IHC	Upregulated in CRC tissues and cell lines.	By activating the JAK/STAT3 pathway	[38]
				Improving cell proliferation and resistance to chemotherapy		
Colorectal cancer	HCT116, SW480, SW1116, LOVO, LS174T	CRC tissues	qRT-PCR, WB, IHC	Upregulated in CRC tissues and cell lines.	By the JAK/STAT3 pathway	[26]
				High expression was associated with depth of invasion, lymphatic node metastasis, distant metastasis, and TNM stage.		
				Knockdown of KIF20A reduced cell proliferation and migration.		
Epithelial ovarian cancer	COV644, COV362, OV90, SKOV3, TOV112D, OVCAR4, A2780, COV434	ovarian cancer tissues	qRT-PCR, WB, IHC	Overexpression related to poorer overall survival and disease progression-free survival.	ND	[31]
				Acted as an independent hazard indicator for predicting clinical outcomes.		
				Overexpression promoted invasion and metastasis and conferred resistance to cisplatin.		
Gastric cancer	AGS, SGC7901, BGC-823, NCI-N87, MGC-803	gastric cancer tissues	qRT-PCR, WB, IHC	Upregulated in Gc tissues.	ND	[32]
				Affected cell proliferation.		
				High expression was related to poor overall survival rate.		
Gastric cancer	SGC-7901	ND	qRT-PCR	Silencing of KIF20A inhibited cell viability and induced G2/M arrest	involved in genistein-induced G2/M arrest of gastric cancer cells	[61]
Glioma	A172, U251, U87	glioma tissues	IHC	Overexpressed in glioma tissues.	ND	[7]
				High expression correlated with poor overall survival.		
				Overexpression promoted cell proliferation and invasion.		
Glioma	U87MG, U251MG, SF126, T98MG, KNS81, KNS42,	glioma tissues	qRT-PCR, WB, IHC	Down-regulated reduced cell proliferation, and induced apoptosis.	Caused failure of cytokinesis and generation of binucleated cells.	[27]
Glioma	ND	ND	ND	MiR-876-3p targeted KIF20A to inhibit glioma cell proliferation, migration, and invasion.	MiR-876-3p inhibited the expression of KIF20A, thus blocking the protein kinase JAK2/STAT3 pathway, and suppressed glioma cell proliferation, migration, and invasion.	[52]
Glioma	ND	ND	ND	Circ-Serpine2 upregulated KIF20A expression, accelerated the proliferation, invasion, and migration of glioma cells, and inhibited apoptosis.	CircRNA-Serpine2 upregulated KIF20A expression by sponging miR-124-3p	[58]
Hepatocellular carcinoma	HepG2, HCC-LM3,	HCC tissues	WB, IHC	Inhibition of KIF20A reduced HCC growth.	As a downstream target of Gli2	[28]
Hepatocellular carcinoma	HepG2/metR	ND	qRT-PCR, IF	KIF20A-depleted cells led to less lung metastasis	As the targert of lactate induced E2F1 to promote carcinogenesis, migration and metastasis of gastric cancer cells	[48]
Hepatocellular carcinomas	HuH6, HuH7, PLC/PRF/5, HepG2, and Hep3B	HCC tissues	qRT-PCR, WB, IHC	Overexpressed in hepatoma tissues and cell lines.	ND	[29]
				Promoted both normal and pathologic hepatocyte proliferation and is linked to tumor aggressiveness.		
Leukemia	HL60	ND	qRT-PCR, WB, IHC	High expression in various human leukemia cell lines and normal bone marrow CD34-positive cells.	ND	[85]
				Knockdown caused accumulation of the cell cycle at G2/M phase and abnormal nuclear division.		
Lung adenocarcinoma	H1975, A549, HCC827	Lung adenocarcinoma tissues	qRT-PCR, WB, IHC	High expressed in soft tissue sarcoma cell lines.	ND	[86]
				Down-regulation inhibited cell proliferation, promoted apoptosis.		
Medulloblastoma	Daoy	Medulloblastoma tissues	IHC	Overexpressed in medulloblastoma tissues.	ND	[33]
				High expression was associated with poor survival.		
Melanoma	CRL1579, 888mel, SK-MEL-19, 164mel, Colo38, HMV-1, SK-MEL-28, Ihara, MEWO, 526mel	Primary melanoma, metastatic melanoma and naevus	qRT-PCR, WB, IHC	Overexpressed in melanoma tissues and cell lines.	ND	[34]
				High expression correlated with cancer metastasis and poor elapse-free survival.		
Melanoma	ND	Melanoma tissues	IHC	High expressed in melanoma.	ND	[21]
Melanoma	A375, A875, MeWo	Melanoma tissues	qRT-PCR, WB, IHC	Overexpressed in melanoma tissues and cell lines.	ND	[87]
Nasopharyngeal carcinoma	CNE1, CNE2, HK1, SUNE1, CNE-2 subclones S18, S26, SUNE1	NPC tissues	qRT-PCR, WB, IHC	Overexpressed in pancreatic cancer tissues and cell lines.	ND	[35]
				High expression was associated with poor survival and prognosis.		
				Knock down suppressed migration and invasion in cell lines.		
Ovarian clear-cell carcinoma	ES-2, TOV-21G, RMG-I, RMG-II, SKOV3, OV-90, KOC-7C	Ovarian CCC tissues	WB, IHC	High expression showed poorer progression-free survival (PFS) and overall survival (OS).	ND	[36]
Pancreatic cancer	PANC1	Pancreatic cancer tissues	RT-PCR, WB, IHC	Overexpressed in pancreatic cancer tissues and cell lines.	ND	[63]
Pancreatic cancer	Panc-1, SU86.86, and T3M4	Chronic pancreatitis and pancreatic ductal adenocarcinoma (PDAC) tissues	qRT-PCR, IHC	Overexpressed in pancreatic cancer tissues and cell lines.	ND	[88]
				Silencing resulted in an inhibition of proliferation, motility, and invasion of pancreatic cancer cell lines.		
Pancreatic cancer	MIA-Paca2	PDAC tissues	qRT-PCR, IHC	Overexpressed in PDAC tissues.	Interacted with DLG5 and effected trafficking system.	[39]
				Down-regulation of KIF20A inhibited growth of PDAC Cells.		
Pancreatic cancer	S2-013, SUIT-2	ND	ND	Down-regulated in S2-013 cells were significantly less invasive.	KIF20A-mediated trafficking of IGF2BP3-containing stress granules and modulation of the motility and invasiveness in pancreatic. Cancers	[41]
Prostate adenocarcinoma	ND	Precancerous tissues	qRT-PCR, IHC	Overexpressed in precancerous tissues.	ND	[89]
Prostate cancer	ND	Prostate cancer tissues	qRT-PCR, WB, IHC	High expression was correlated with adverse clinicopathological features.	ND	[90]
				Knocking down suppressed the proliferation, migration, and invasion of the prostate cancer cell.		
Renal carcinoma	OS-RC-2, 769-P, CaKi-1, UM-RC-2 and 786-O	ND	qRT-PCR, WB	KIF20A could partially reverse the inhibition of IRF6 caused on the proliferation, invasion, migration and metastasis of renal carcinoma cells.	KIF20A was a target of IRF6.	[59]
Renal clear cell carcinoma	OSRC-2, SW839, Caki-1, A498	ccRCC tumor samples	qRT-PCR, WB	Up-regulated in ccRCC tissue.	ND	[91]
				Promoted proliferation and invasion, inhibits the apoptosis of renal cancer cells		
Soft tissue sarcoma	WEHI164, MCA101, MCA207	ND	qRT-PCR, WB	High expressed in soft tissue sarcoma cell lines.	ND	[92]
				Down-regulation inhibited cell proliferation, migration and invasion, promoted apoptosis.

**Fig. 4:**
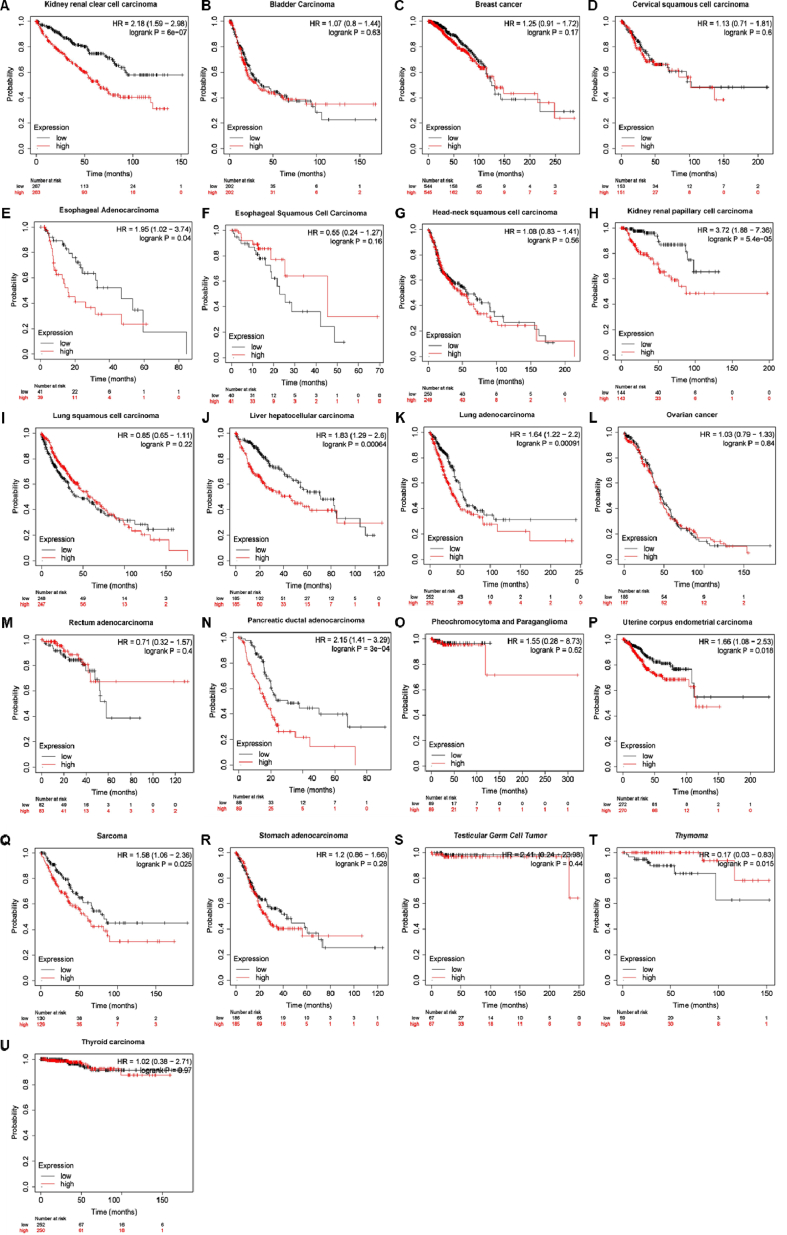
High expressed KIF20A predicted a poor OS. Data from TCGA databases. A. Kidney renal clear cell carcinoma. B. Bladder Carcinoma. C. Breast cancer. D. Cervical squamous cell carcinoma. E. Esophageal Adenocarcinoma. F. Esophageal Squamous Cell Carcinoma. G. Head-neck squamous cell carcinoma. H. Kidney renal papillary cell carcinoma. I. Lung squamous cell carcinoma. J. Liver hepatocellular carcinoma. K. Lung adenocarcinoma. L. Ovarian cancer. M. Rectum adenocarcinoma. N. Pancreatic ductal adenocarcinoma. O. Pheochromocytoma and Paraganglioma. P. Uterine corpus endometrial carcinoma. Q. Sarcoma. R. Stomach adenocarcinoma. S. Testicular Germ Cell Tumor. T. Thymoma. U. Thyroid carcinoma.

**Fig. 5:**
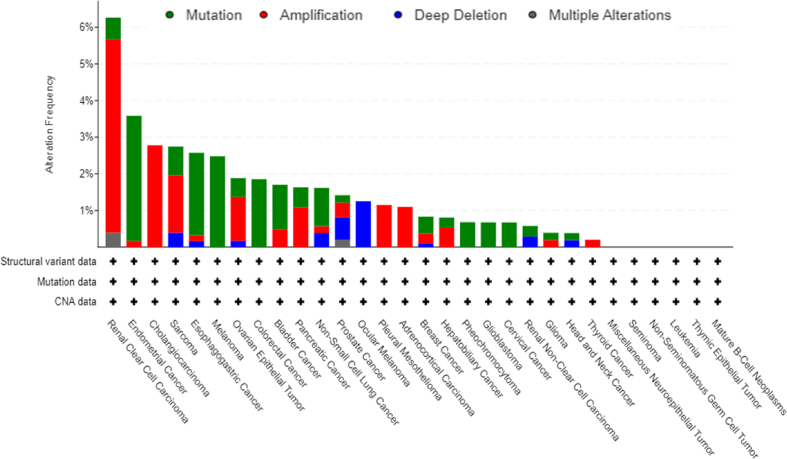
KIF20A genome was altered in multiple cancers. Data from http://www.cbioportal.org/.

High expression of KIF20A was associated with poor OS [21]. In breast cancer, KIF20A predicted a poor prognosis and was an independent prognostic factor [25], as well as in bladder cancer [24], cervical squamous cell carcinoma [30], epithelial ovarian cancer [31], gastric cancer [32], glioma [7], medulloblastoma [33], melanoma [34], nasopharyngeal carcinoma [35], and ovarian clear-cell carcinoma [36]. Besides, there were plenty of studied predicted that KIF20A was highly expressed in cancers and associated with poor OS based in public data platforms, for example, TCGA, GEO (Table 2). We also used TCGA databases to predict the relationship of KIF20A and OS (Fig. 4A-U), results showed that KIF20A was negatively related to OS. Besides, Moreover, changes in the KIF20A genome were also associated with cancers (Fig. 5, data was from cBioPortal). All of the studies suggested that KIF20A could be a prognostic biomarker.

**Table 2:** KIF20A worked as a tumor-associated antigen.

Tumor	Clinical trial	Peptides combination therapy	Results	Reference
**Biliary tract cancer**	phase I	cell division cycle associated 1 (CDCA1), cadherin 3 (CDH3)	Peptide vaccines were well tolerated, and no serious adverse events were observed after vaccination. Peptide specific T cell immune response was also observed in all patients.	[68]
**Biliary tract cancer**	ND	VEGFR1, VEGFR2	Four out of six patients exhibited vaccine-specific T-cell responses. Vaccine-specific T cell responses contributed significantly to overall survival.	[69]
**Gastric cancer**	ND	DEPDC1, URLC10, FoxM1, and VEGFR1	Patients who showed T cell responses specific to antigen peptides had a tendency towards better survival. The Patients with local skin reactions had significantly better OS	[73]
**Gastric cancer**	ND	DEPDC1, URLC10, FOXM1, and VEGFR1, combined with S-1	No adverse events of grade 3 or higher were observed.	[74]
**Gliomas**	ND	LY6K, DEPDC1, FOXM1, VEGFR1 and VEGFR2	The treatment was well tolerated, induced immunoreactivity, and contributed to overall survival.	[76]
**Head-and-neck malignant tumor**	ND	ND	KIF20A specific Th1 cell response was detected after short-term stimulation of PBMC.	[67]
**Pancreatic cancer**	ND	ND	KIF20A peptide induced CTL with peptide specific cytotoxicity.	[63]
**Pancreatic cancer**	phase I/II	ND	OS time of the patients was significantly prolonged.	[65]
**Pancreatic cancer**	phase I	ND	Patients was well tolerated. No serious adverse events were observed. Peptide specific T cell responses were detected.	[66]
**Pancreatic cancer**	phase I	gemcitabine	No serious adverse reactions related to KIF20A peptide were observed. Of the 9 patients who completed at least one course of treatment, 4 of the them were induced to produce IFN-g cells, and 4 were stable.	[70]
**Pancreatic cancer**	Phase II	VEGFR1 and VEGFR2	Peptide-specific CTL induction for KIF20A or VEGFR1 showed a better prognosis. Patients who showed a strong injection site reaction had a better survival rate	[72]
**Pancreatic cancer**	Phase II	VEGFR1 and VEGFR2 combined with gemcitabine	Vaccine was well tolerated and induced KIF20A-specific CTL responses. All four patients who underwent R0 resection with KIF20A expression had no recurrence of pancreatic cancer with KIF20A-specific CTL responses. Vaccine contributed to overall survival.	[71]
**Pediatric refractory solid tumors**	ND	KOC1 and FOXM1	Induce a sufficient number of peptide specific CTLs, contributed to free survival.	[75]

### 2.2 The mechanisms of KIF20A in regulating multiple tumors

KIF20A regulated cancer through cell cycle. In breast cancer, suppressing endogenous KIF20A using small interfering ribonucleic acids or paprotrain, a specific inhibitor of KIF20A, significantly inhibited breast cancer cell growth. Further results indicated that KIF20A arrested cells at the G2/M phase, caused mitotic cell death [25]. In glioma cancer cells, down-regulated KIF20A caused failure of cell division and binucleated cell generation, resulting in decreased cell proliferation and increased cell apoptosis [27] (Fig. 2C).

**Fig. 6:**
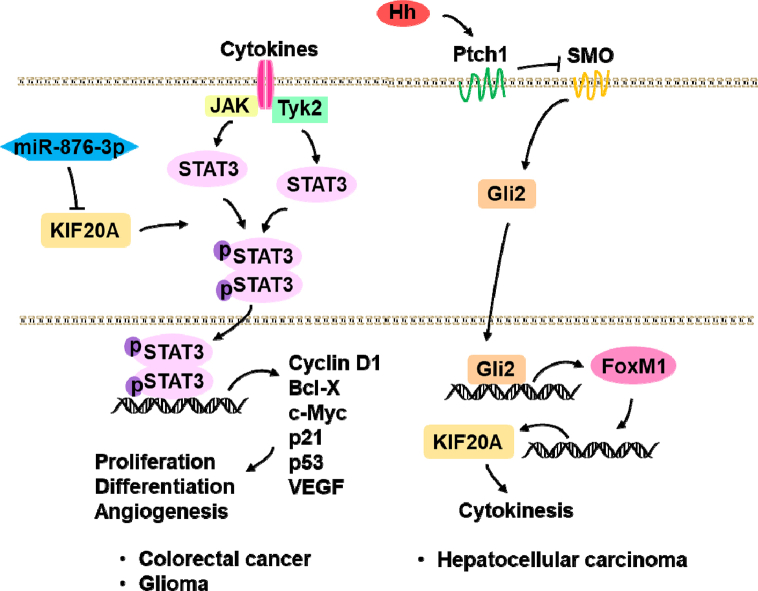
KIF20A regulated cancer via JAK-STAT3 and Hh signaling pathways. In colorectal cancer, increased KIF20A promoted cell proliferation through activated JAK-STAT3; in glioma, miR-876-3p suppressed KIF20A, inhibited JAK2-STAT3 signaling pathway; in hepatocellular carcinoma, KIF20A was a target of Hh signaling, Gli2 enhanced KIF20A by FoxM1, increased KIF20A promoted HCC growth and survival.

Janus kinase (JAK)-signal transducer and activator of transcription (STAT) 3 signaling pathway drives proliferation, survival, invasiveness, and metastasis of cancer cells [37]. In colorectal cancer, increased KIF20A promoted cell proliferation [38], decreased KIF20A reduced cell proliferation and migration [26]. Besides, high expression of KIF20A was associated with invasion, lymphatic node metastasis, distant metastasis, and TNM stage. Activated JAK-STAT3 pathway was involved in these processes [26] (Fig. 6).

KIF20A and discs large MAGUK scaffold protein 5 (DLG5) were colocalized in PDAC cells [39]. DLG5 formed a complex with vinexin and β-catenin and was involved in cell-cell connection [40]. The transport of DLG5 to certain sites was important [39]. DLG5 was a cargo of KIF20A. Knockdown KIF20A or DLG5 suppressed the proliferation of PDAC cells [39], indicating that down-regulated KIF20A attenuated growth by reducing transport of DLG5. In another study, KIF20A transported RNA-binding protein IGF2BP3 and IGF2BP3-bound transcripts toward cell protrusions along microtubules [41]. IGF2BP3 bound mRNAs, such as ADP ribosylation factor 6 (Arf6) and Rho guanine nucleotide exchange factor 4 (Arhgef4), were subsequently translated in membrane processes, in turn, these locally translated proteins increased invasiveness and metastasis of PDAC cells. Thus, KIF20A mediated IGF2BP3 transport, promoted motility and invasiveness in pancreatic cancer [42].

### 2.3 The regulation of KIF20A by proteins and non-coding RNAs

KIF20A was highly expressed in multiple cancer; high expression of KIF20A indicated a poor OS. Also, KIF20A can be a target of a variety of proteins and non-coding RNAs (Table 1).

Glioma-associated oncogene 2 (Gli2) is transcriptional regulator involved in Hedgehog (Hh) signaling, and is essential for HCC growth and survival [43,44]. Gli2 was overexpressed in HCC [28], knockdown of Gli2 inhibited HCC cell growth [45], and Gli2 was involved in the direct regulation of key cell cycle regulators in G1 phase [46]. KIF20A was a downstream target of Hh signaling, besides, Gli2 enhanced KIF20A expression by activating forkhead box protein M1 (FoxM1) [28]. Gli2-KIF20A axis played a key role in HCC growth and survival (Fig. 6).

Due to Warburg Effect, a large amount of lactate accumulated in the tumor microenvironment. In turn, the accumulated lactate promoted tumor progression [47]. In a lactate-enriched environment in HepG2 cells, bioinformatic analysis explored the key in lactate induced cell motility regulation. Results suggested that kinesin family genes might play an important role, including KIF20A. Further results proved that lactate induced the expression of E2F1, E2F1 regulated microtubule dynamics to promote lactate-dependent cell motility through kinesin proteins [48]. Thus, accumulated lactate induced the expression of E2F1, E2F1 regulated KIF20A to promote carcinogenesis, migration and metastasis of HepG2/metR cells.

**Fig. 7:**
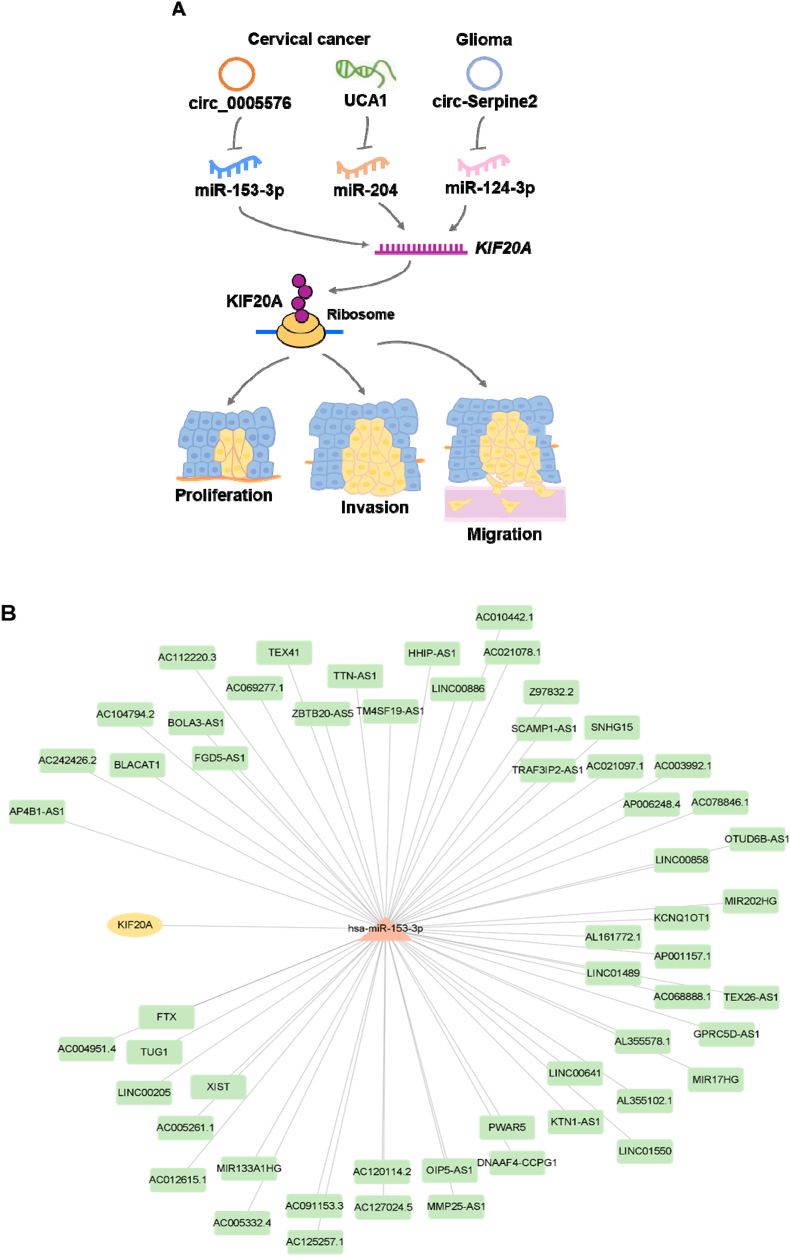
KIF20A was a target of circRNA in facilitating cancer. A. In cervical cancer, circ_0005576 and UCA1 acted as sponges of miR-153-3p and miR-204 to increase KIF20A; in glioma, circRNA-Serpine2 upregulated KIF20A expression by sponging miR-124–3p. Increased KIF20A accelerated the proliferation, invasion, migration, and inhibited apoptosis of cancer cells. B. lncRNA-miRNA-KIF20A network. The KIF20A-miRNA targets were predicted with TargetScanHuman (https://www.targetscan.org/vert_71/) and the miRNA-lncRNA targets were predicted with ENCORI (https://www.targetscan.org/vert_71/), the network was generated with Cytoscape (v 3.9.1).

Circular RNA (circRNA) is a kind of noncoding cancer genome. It is involved in tumor occurrence and can be used as a biomarker for diagnosis and prognosis, as well as a possible therapeutic target in personalized medicine [49]. Circ_0005576 was overexpressed in both cervical cancer tissues and cell lines, positively associated with advanced stages, lymph node metastasis, but negatively related to OS. Knockdown of circ_0005576 suppressed the growth, colony formation and metastasis of cervical cancer cells. Circ_0005576 was located in the cytoplasm and acted as a sponge of miR-153-3p to increase KIF20A. KIF20A was a target of circ_0005576 in facilitating cervical cancer progression [50] (Fig. 7A). Coincidentally, miR-153-3p was included in the miRNAs and lncRNAs we predicted to be associated with KIF20A (Fig. 7B).

MicroRNAs (miRNA) are the key in human cancer progression, and can be therapeutic targets [51]. MiR-876-3p was significantly downregulated in glioma tissues and cell lines. Overexpression of miR-876-3p suppressed cell proliferation, epithelial-mesenchymal transition (EMT), migration, and invasion, and inhibited the expression of KIF20A and activation of JAK2/STAT3 signaling pathway [52]. In the treatment of glioma, KIF20A could become the target of miR-876-3p (Fig. 6).

Urothelial cancer associated 1 (UCA1) is a member of the long non-coding RNA genes. Previous studies indicated that UCA1 was a tumor biomarker of bladder adenocarcinoma [53], participated in breast cancer [54], colorectal cancer [55], and gastric cancer [56]. In cervical cancer tissues and cell lines, UCA1 was highly expressed, knocked down UCA1 inhibited proliferation and invasion. Further analysis revealed that miR-204 was a target of UCA1, UCA1 sponged miR-204 thus increased KIF20A in cervical cancer. Thus, UCA1 upregulated KIF20A expression to exacerbate cervical cancer via sponging miR-204 [57] (Fig. 7A).

In glioma-derived exosomes, bioinformatics analysis revealed that KIF20A was one of the hub genes. CircRNA-miRNA-mRNA network suggested that circRNA-Serpine2 upregulated KIF20A expression by sponging miR-124-3p. Increased KIF20A accelerated the proliferation, invasion, migration, and inhibited apoptosis of glioma cells [58] (Fig. 7A).

Interferon regulatory factor (IRF) 6 was down-regulated in renal carcinoma, decreased interferon regulatory factor (IRF) 6 indicated a poor OS and disease-free survival. High expression of IRF6 inhibited proliferation, invasion, migration and metastasis of renal carcinoma cells. Interestingly, KIF20A could partially reverse the effects of IRF6 on renal carcinoma cells. ChIP experiment verified the combination of IRF6 and KIF20A promoters, indicating that KIF20A was a target of IRF6 in renal carcinoma [59].

### 2.4 KIF20A acts as a therapeutic target for cancer

#### 2.4.1 KIF20A works as a target in anti-cancer treatment

Genistein plays an anti-cancer role by inducing G2/M phase arrest and apoptosis [60]. After treatment of genistein, gastric cancer cells were arrested in G2/M phase, the expression of KIF20A was reduced [61]. KIF20A had been proved to be involved in G2/M phase arrest [14], and played an important role in gastric cancer [32]. Genistein might inhibit gastric cancer by downregulating KIF20A [61] (Fig. 2C).

#### 2.4.2 Identified KIF20A as a tumor-associated antigen

In anti-cancer immunotherapy, cytotoxic T lymphocytes (CTLs) play an absolutely key role. CTLs, also known as CD8^+^ T cells or killer T cells, are a kind of T cell subset that kill target cells expressing specific antigens. CTLs kill tumors by recognizing tumor associated antigen (TAA) combined with major histocompatibility complex I (MHC I). They are the main effector cells of anti-tumor immunity [62].

KIF20A was weakly expressed in testis and thymus, highly expressed in pancreatic cancer, skin, peritoneal metastases, and other human leukocyte antigen (HLA)-A2 positive cancer cell lines. Thus, KIF20A seems to be a perfect TAA (Table 2). HLA-A2 transgenic mice (Tgm) were used to identified KIF20A-derived and HLA-A2-restricted mouse CTL epitopes, KIF20A-2, KIF20A-8 and KIF20A-28 could induce HLA-A2-restricted CTLs in HLA-A2 Tgm without autoimmunity. Then, these three peptides were used to stimulate CD8^+^T cells isolated from PBMC of HLA-A2 positive healthy donors, as a results, KIF20A peptides induced CTL with specific cytotoxicity, peptides were naturally processed and expressed on the surface of cancer cells. KIF20A was a promising tumor-associated antigen in the treatment of pancreatic cancer [63].

In another study of pancreatic cancer, it was proved that KIF20A-10-66 peptide was a new HLA-A24 restricted tumor associated antigen, and can be used in CTL induced tumor therapy [64]. Based on this study, a phase I/II clinical trial was conducted to confirm the efficacy of KIF20A-66 (KIF20A-10–66) peptide in immunotherapy for patients with advanced pancreatic cancer (clinical trial registration: UMIN-CTR, number UMIN000004919). In this study, KIF20A-66 peptide was subcutaneously injected into metastatic pancreatic cancer patients who failed to treate with gemcitabine. Results showed patients had good tolerance. Among the 29 patients who completed at least one course of treatment, 21 patients were stable, 8 patients were progressive, and 8 patients had reduced tumors, of which 1 was completely relieved, the OS of patients was significantly prolonged. Therefore, KIF20A-66 vaccine was effective for advanced pancreatic cancer [65].

Another phase I clinical trial used 4 peptides as vaccine in advanced pancreatic cancer, including KIF20A. This clinical trial enrolled 9 patients with advanced pancreatic cancer who failed to respond to standard chemotherapy. Patients received subcutaneous vaccination every week, the polypeptide vaccine was well tolerated. No serious adverse events were observed after vaccination. Peptide specific T cell responses were detected in all 9 patients, of which 4 patients observed clinical benefits [66].

Previous studies focused on CTL, but there were also studies on Th cells. First, computer was used to predict a long peptide (LP) of KIF20A which could candidate promiscuous Th1-cell epitopes containing CTL epitopes, then LP was used to stimulate peripheral blood mononuclear cells (PBMC) derived from healthy donors or patients with head-and-neck malignant tumor (HNMT). HLA-A24 transgenic mice were used to verify whether vaccination with KIF20A-LP induces efficient cross-priming of CTLs in vivo. Finally, KIF20A-LP bearing naturally processed epitopes recognized by CD4^+^T cells and CTLs were identified. After injected CTL induced KIF-20A-LPs, expression of KIF20A was detected in 55% of HNMT, KIF20A specific Th1 cell response was detected after short-term stimulation of PBMC in 50% of HNMT patients, indicated that KIF20A-LP can induce tumor-specific Th1 cells and CTLs at the same time [67].

In advanced biliary tract cancer (BTC), a phase I clinical trial (UMIN-CTR000003229) was also carried out with KIF20A peptide. Nine patients with advanced BTC were selected and injected with three peptides, including KIF20A. Results showed that three peptide vaccines were well tolerated, no serious adverse events were observed after vaccination. Peptide specific T cell immune response was observed in all patients, and 5 of the 9 patients were stable, indicating that KIF20A peptide was effective in BTC [68]. Another similar study came to the same conclusion [69].

A phase I clinical trial combining KIF20A derived peptide with gemcitabine (GEM) was conducted in patients with advanced pancreatic cancer who had received chemotherapy and/or radiotherapy. In this clinical trial, no serious adverse effect related to KIF20A peptide were observed. IFN-γ-producing cells were induced in four out of nine patients who completed at least one course of treatment [70]. Another two similar clinical trials verified these results [71,72], suggested that this combination therapy was promising for advanced pancreatic cancer.

In advanced gastric cancer (GC), a clinical trial using HLA-A24 binding peptide vaccine verified the effectiveness. In 35 GC patients, a polypeptide mixture vaccine, including KIF20A were injected, 4 patients developed severe skin reactions. The patients with local skin reaction had a better OS. Peptide vaccine therapy has been found to be safe and is expected to induce specific T cell responses in patients with advanced GC [73]. Combined this cancer vaccine therapy with S-1 chemotherapy, it was tolerable [74].

In pediatric refractory solid tumors, multiple peptides mixtures were injected. The clinical response of this trial showed that 4 patients were stable after 8 weeks, and 2 patients were in remission for more than 11 months. KIF20A induced a sufficient number of peptide specific CTLs, and these patients had better progression free survival. This study provided strong evidence for the effectiveness of KIF20A as a tumor vaccine [75].

In the study of glioma, ten patients received subcutaneous injection of mixtures including KIF20A peptide. The treatment was well tolerated without any serious systemic adverse events. In all six assessable patients, the vaccine induced immune responses. Median OS was 9.2 months. Five patients achieved progression free status for at least six months. Two patients with recurrent glioblastoma were stable. One patient with anaplastic oligoastrocytoma achieved complete remission 9 months after inoculation. In conclusion, the treatment was well tolerated and effective [76].

#### 2.4.3 KIF20A in tumor chemotherapy

KIF20A not only increased in tumors, related to OS, but also took part in tumor drug resistance. FOXM1 is a transcription factor which plays an important role in cell cycle. Previous studies found that FOXM1 was highly expressed and involved in genotoxic agent-resistant cancer cells, but the mechanism was not clear [77,78]. To explore the role of FOXM1 in paclitaxel treatment, *FoxM1*-deleted breast cancer line MEFs were used, results showed that cell viability was decreased and cellular senescence was increased in response to paclitaxel treatment [79]. KIF20A was a target of FOXM1 in normal spindle formation and chromosome segregation [80]. Downregulation of FOXM1 decreased KIF20A. FOXM1 regulated KIF20A to modulate paclitaxel sensitivity in breast cancer [79], as well as in docetaxel resistance of prostate cancer [81]. FOXM1 knockdown induced cell apoptosis and G2/M cell cycle arrest, suppressing cell migration and invasion in docetaxel-resistant prostate cancer cell lines. FOXM1 inhibitor thiostrepton significantly weakened docetaxel resistance. FOXM1 and KIF20A exhibited consistent and highly correlated overexpression in prostate cancer cells and tissues, highly expressed FOXM1 may help promote docetaxel resistance by inducing KIF20A expression [81].

Pimozide could be a promising drug to overcome taxane cabazitaxel (CBZ) resistance in docetaxel-resistant prostate cancer (CRPC) patients by targeting AURKB and KIF20A [82]. CBZ is a promising treatment for CRPC, however, it has limited on prolonging survival. Pimozide was a promising candidate drug for CBZ-resistant CRPC, and had a significant anti-tumor effect. Microarray analysis identified AURKB and KIF20A as potential targets of pimozide in CBZ-resistant CRPC. AURKB and KIF20A were highly expressed in cabazitaxel-resistant prostate cancer cells. Pimozide suppressed KIF20A mRNA expression, moreover, KIF20A expression was suppressed by PZD administration in mouse model, indicating that pimozide could overcome CBZ resistance in CRPC through KIF20A [82].

Induction of ferroptosis significantly reversed oxaliplatin resistance of colorectal cancer [83]. KIF20A was highly expressed in the oxaliplatin-resistant cell lines and strongly correlated with survival among colorectal cancer patients. Knockdown of KIF20A enhanced the sensitivity of colorectal cancer cells to oxaliplatin and inhibited the activation of NUAK1, a kinase related to malignant progression and poor prognosis of CRC [84]. NUAK1 agonist reversed the effect of KIF20A on oxaliplatin. In addition, knockdown of NUAK1 upregulated PP1β, suppressed the phosphorylation of GSK3βSer9, inhibited Nrf2 transferring into the nucleus, decreased the expression of feroptosis key negative regulatory protein (GPX4), and abolished cell resistance. The effect of NUAK1 on oxaliplatin could also be reversed by using Nrf2 agonist. Therefore, KIF20A/NUAK1/PP1β/GPX4 pathway might inhibit ferroptosis, which are important in CRC resistance to oxaliplatin [83].

## 3 Conclusion and future perspectives

KIF20A was lowly expressed in normal tissues and highly expressed in tumors; high expression of KIF20A indicated a poor OS. Decreased KIF20A suppressed cancer cell proliferation, migration and invasion. Many mechanisms were involved in KIF20A promoting tumors. As a member of kinesin family, KIF20A took part in the progression of cancers through regulating cell division. Also, JAK-STAT signaling pathway and some molecules like Gli2, DLG5 and IGF2BP3 were involved in. At the same time, KIF20A was the target of circRNAs and miRNAs. KIF20A was related to drug or chemotherapy resistance in tumor treatment. It seems that KIF20A could be a promising target for cancer.

There are a lot of studies exploring whether KIF20A could be a therapeutic target for cancer. The most studied was to make KIF20A peptides into tumor vaccine. Both phase I and phase II clinical trials have shown that KIF20A vaccine could alleviate tumors, especially in pancreatic cancer. These results confirmed that KIF20A is a promising therapeutic target of multiple cancer.

## Declarations

### Author contribution statement

All authors listed have significantly contributed to the development and the writing of this article.

### Funding statement

Zheng Jin was supported by 10.13039/501100021171Guangdong Basic and Applied Basic Research Fund [No. 2021A1515110975], 10.13039/501100012271Shenzhen Science and Technology Program [No. JCYJ20220530151211025]. Dr Zhenhua Zhu was supported by Guangzhou Basic and Applied Basic Research Fund [SL2023A04J00435]. Damo Xu was supported by 10.13039/501100012234Shenzhen Peacock Plan Team Project [No. 201703313000321].

### Data availability statement

No data was used for the research described in the article.

## Declaration of competing interest

The authors declare no competing interests.
